# Platform Comparison for Evaluation of ALK Protein Immunohistochemical Expression, Genomic Copy Number and Hotspot Mutation Status in Neuroblastomas

**DOI:** 10.1371/journal.pone.0106575

**Published:** 2014-09-04

**Authors:** Benedict Yan, Chik Hong Kuick, Malcolm Lim, Kavita Venkataraman, Chandana Tennakoon, Eva Loh, Derrick Lian, May Ying Leong, Manikandan Lakshmanan, Vinay Tergaonkar, Wing-Kin Sung, Shui Yen Soh, Kenneth T. E. Chang

**Affiliations:** 1 Department of Pathology and Laboratory Medicine, KK Women's and Children's Hospital, Singapore, Singapore; 2 Saw Swee Hock School of Public Health, National University of Singapore and National University Health System, Singapore, Singapore; 3 Genome Institute of Singapore, Singapore, Singapore; 4 Mouse Models for Human Cancer Unit, Institute of Molecular and Cell Biology, Singapore, Singapore; 5 School of Computing, National University of Singapore, Singapore, Singapore; 6 Haematology/Oncology Service, Department of Paediatric Subspecialties, KK Women's and Children's Hospital, Singapore, Singapore; CCR, National Cancer Institute, NIH, United States of America

## Abstract

ALK is an established causative oncogenic driver in neuroblastoma, and is likely to emerge as a routine biomarker in neuroblastoma diagnostics. At present, the optimal strategy for clinical diagnostic evaluation of ALK protein, genomic and hotspot mutation status is not well-studied. We evaluated ALK immunohistochemical (IHC) protein expression using three different antibodies (ALK1, 5A4 and D5F3 clones), *ALK* genomic status using single-color chromogenic in situ hybridization (CISH), and *ALK* hotspot mutation status using conventional Sanger sequencing and a next-generation sequencing platform (Ion Torrent Personal Genome Machine (IT-PGM)), in archival formalin-fixed, paraffin-embedded neuroblastoma samples. We found a significant difference in IHC results using the three different antibodies, with the highest percentage of positive cases seen on D5F3 immunohistochemistry. Correlation with *ALK* genomic and hotspot mutational status revealed that the majority of D5F3 ALK-positive cases did not possess either *ALK* genomic amplification or hotspot mutations. Comparison of sequencing platforms showed a perfect correlation between conventional Sanger and IT-PGM sequencing. Our findings suggest that D5F3 immunohistochemistry, single-color CISH and IT-PGM sequencing are suitable assays for evaluation of ALK status in future neuroblastoma clinical trials.

## Introduction

ALK is an established causative oncogenic driver in neuroblastomas. With a recent phase 1 trial documenting complete response to the ALK inhibitor crizotinib in two patients with neuroblastomas [1], it seems probable that ALK status (whether protein, genomic or both) will emerge as a routine biomarker in neuroblastoma diagnostics. Against this backdrop, we performed a study evaluating the various platforms for ALK protein and genomic status characterization.

ALK protein expression in neuroblastomas has been reported as an adverse prognostic factor by several groups [2]–[5]. At present, with regards to ascertainment of ALK immunohistochemical status, the major unresolved issue appears to be the choice of antibody, there being several available commercially (Table 1). In our earlier analysis, we observed ALK expression in only 1/54 (1.85%) of neuroblastomas [6]. This contrasts sharply with the aforementioned studies, in which the prevalence of strong ALK immunohistochemical expression is generally around 50% or higher [2]–[5]. We were thus interested in comparing the performance of different antibodies.

**Table 1:** Commercially available monoclonal ALK antibodies.

Clone	Tumor type studied	Company	Reference
ALK1	Neuroblastoma	DAKO Denmark A/S, Glostrup, Denmark	De Brouwer 2010 [2]; Yan 2013 [6]
D5F3	Lung adenocarcinoma	Cell Signaling, Danvers, MA, USA	Ying 2013 [37]
RM-9108	Neuroblastoma	Thermo Fisher Scientific, Fremont, CA, USA	Duijkers 2012 [3]
5A4	Neuroblastoma	Thermo Fisher Scientific, Fremont, CA, USA	Passoni 2009 [4]

High-level *ALK* genomic amplification, although infrequent (prevalence 5% or less), has also been reported as an adverse prognostic factor in neuroblastomas in some studies [5], [7], [8]. We evaluated the performance of a chromogenic in situ hybridization (CISH) assay [9] for ascertaining *ALK* copy number status.

*ALK* sequence mutations have been reported in approximately 5–10% of neuroblastomas [7], [10]–[12], and these predominantly occur within the tyrosine kinase domain, the two hotspots being p.F1174 and p.R1275 [13]. The presence of *ALK* mutations appears to have clinical implications. For example, the presence of the p.F1174L mutation was associated with a worse prognosis in *MYCN*-amplified neuroblastomas in one study [2], and resistance to crizotinib in another preclinical *in vitro* study [14].

Although Sanger sequencing is considered the gold standard for mutational analysis [15], [16], there has been considerable interest in the use of next-generation sequencing (NGS) platforms for clinical diagnostics [17], [18]. To date, NGS analysis has been applied to neuroblastomas predominantly for discovery work [19]–[22]. From a clinical quality management perspective, we were interested to compare the performance of the Ion Torrent Personal Genome Machine (IT-PGM), a benchtop NGS platform, to Sanger sequencing in the detection of *ALK* mutations occurring at the p.F1174 and p.R1275 hotspots.

## Materials and Methods

### Study population and clinicopathological data

A total of 118 neuroblastoma samples (comprising 36 pre-treatment, 53 post-treatment, 26 relapsed or metastatic samples, and 3 with unknown treatment status) from 95 patients, were identified from the archives of the Department of Pathology and Laboratory Medicine, KK Women's and Children's Hospital, Singapore. Clinicopathological data including mean age at first biopsy, gender, stage and *MYCN* amplification status was extracted (Table 2).

**Table 2:** Clinicopathological characteristics of study cohort.

Age at first biopsy (yrs, N = 95)	
Mean (Range)	3.54 (0.02–10.94)
**Gender (N = 95)**	
Male	60
Female	35
**Sample type (N = 118)**	
Pre-treatment primary tumors	36
Post-treatment primary tumors	53
Relapsed/metastatic tumors	26
Treatment status unknown	3
**Histology (N = 118)**	
Neuroblastoma	67
Ganglioneuroblastoma	16
Ganglioneuroma	3
Neuroblastic tumor, unspecified	32
***MYCN*** ** amplification status (N = 108)**	
Amplified	26
Non-amplified	81
Equivocal	1

### Ethics Statement

Ethics approval was obtained from the Singhealth Centralised Institutional Review Board (CIRB Ref: 2012/450/B).

### Tissue microarray construction

Tissue microarray construction (TMA) was performed for 112 samples. One tissue core (1.0 mm diameter) was punched from a representative tumor area from the donor tissue blocks and deposited into a recipient block using a manual tissue-arraying instrument (Beecher Instruments, Sun Prairie, Wisconsin, USA).

### Immunohistochemistry

The following anti-ALK antibodies were used: ALK1 antibody (Dako Denmark A/S, Glostrup, Denmark), D5F3 antibody (Cell Signaling, Danvers, MA, USA) and 5A4 antibody (Leica Biosystems, Newcastle Upon Tyne, UK). Immunohistochemical staining was performed using the dilutions and platforms as stated in Table 3.

**Table 3:** Antibodies used in this study with corresponding platforms.

Clone (dilution)	Company	Platforms	Antigen Retrieval
ALK1 (1∶25)	DAKO Denmark A/S, Glostrup, Denmark	Ventana Benchmark ULTRA (Ventana Medical Systems, AZ, USA)	CC1 solution (Ventana Medical Systems, AZ, USA) for 36 min
D5F3 (1∶100)	Cell Signaling, Danvers, MA, USA	Ventana Benchmark XT (Ventana Medical Systems, AZ, USA)	CC1 solution (Ventana Medical Systems, AZ, USA) for 64 min
5A4 (1∶60)	Leica Biosystems, Newcastle Upon Tyne, UK	Ventana Benchmark XT (Ventana Medical Systems, AZ, USA)	CC1 solution (Ventana Medical Systems, AZ, USA) for 64 min

Scoring (taking into account staining intensity and percentage positivity, modified from Passoni et al [4]) was performed as follows: 0, no staining; 1+, weak cytoplasmic staining, >1% of cells; 2+, moderate cytoplasmic staining, >50% of cells; 3+, strong cytoplasmic staining, >50% of cells. Similar to guidelines for *HER2* IHC scoring [23], the following categories were defined: 0 and 1+ - negative; 2+ - equivocal; 3+ - positive.

### Chromogenic in situ hybridization

*ALK* genomic copy number status was determined by using the ZytoDot 2C SPEC ALK break-apart probe (ZytoVision, Bremerhaven, Germany). The protocol is similar to that previously reported [9] with some minor modifications. After deparaffinization in xylene and ethanol, the slides were incubated in 3% hydrogen peroxide in methanol for 5 min to quench endogenous peroxidase activity. 15 min incubation in EDTA pretreatment buffer (ZytoVision) at 95°C in a water bath, followed by digestion with pepsin solution (ZytoVision) at RT for 6 min was performed. The sections were dehydrated in graded ethanol and air-dried. 10–20 ml of probe were applied to the tissue, covered with a coverslip, and sealed with rubber cement. The slides were denatured at 79°C for 5 min and hybridized overnight at 37°C in a TDH-500 slide denaturation/hybridization system (Hangzhou Allsheng Instruments Co., Zhejiang, China). After removal of the coverslips, the slides were washed for 5 min in Wash Buffer SSC (ZytoVision) at 75–80°C and rinsed in deionized water for 2 min at room temperature. Probe detection was performed with sequential incubation of rabbit-anti-DNP and anti-rabbit-AP antibody, each for 15 min at 37°C in a humidity chamber, followed by incubation with the color substrates AP-Red for 10 min at room temperature (ZytoVision). The tissue was counterstained with the Nuclear Blue Solution provided by the kit, washed in running tap water for 2 min and dehydrated in a short sequence of 100% ethanol (3×30 s) and xylene (2×30 s) before coverslipping.

Twenty non-overlapping cells were evaluated. Similar to guidelines for *HER2* genomic status [23], the following categories were defined: average *ALK* copy number >6 signals/nucleus - positive; 4–6 signals/nucleus - equivocal; <4 signals/nucleus - negative.

### Cell line-Derived Tumor Xenografts

All the animal experiments were carried out with approval from Institutional Animal Care and Use Committee (IACUC) at the Biological Resource Center, A-STAR, Singapore. 5×10^6^ SKNF1, SH-SY5Y and SK-N-SH neuroblastoma cells (obtained from the American Type Culture Collection) in matrigel (1∶1) were implanted subcutaneously on the flank of four to six old female Balb/c nude mice (BRC, Singapore). Tumor growth was monitored over a period of time and tumor volume (mm^3^) calculated by the formula V = L*B^2^/2, where V = volume of tumor, L = length of tumor and B = breadth of tumor using electronic Vernier callipers (Mitutoyo, Japan). When the tumor volume was between 200–300 mm^3^, the animals were euthanized. The tumors were removed and fixed in 10% neutral buffered formalin solution.

### DNA extraction

DNA was extracted from formalin-fixed, paraffin-embedded (FFPE) samples using ReliaPrep gDNA FFPE kit, Promega. Percentage of tumor cells, as assessed by light microscopy, was >30% in all cases. Purified DNA was quantified using the Qubit (Invitrogen, Life Technologies).

### Sanger sequencing

PCR amplification of *ALK* exons 23 (containing the p.F1174 hotspot) and 25 (containing the p.R1275 hotspot) was performed using the following primers (as reported by Chen et al) [10]: exon 23 forward primer – AAGATTTGCCCAGACTCAGC, exon 23 reverse primer – TGTCCTTGGCACAACAACTG; exon 25 forward primer – TAGTGATGGCCGTTGTACAC, exon 25 reverse primer - CCAGGAGATGATGTAAGGGA. The PCR product was analyzed by routine sequencing using the ABI 3730xl DNA sequencer (Applied Biosystems, Foster City, CA).

### IT-PGM Sequencing

Sequencing was performing according to the manufacturer's protocol using 10 ng DNA, Ion AmpliSeq primer pool and Ion AmpliSeq Library Kit 2.0 Beta (Life Technologies) for sequencing of the *ALK* gene. Briefly, PCR enrichment of 5182 bp of the *ALK* coding region, corresponding to 66 amplicons and 100% of the gene, was performed. Template preparation was performed using the Ion OneTouch system (Life Technologies). The Ion Sphere Particles were recovered and enriched according to the manufacturer's protocol. Samples were loaded onto either a 314 or 316 chip.

### Bioinformatics analysis

Bioinformatics analysis was performed using the vendor-provided data analysis pipeline (Torrent Suite Software version 4.0.2 and Ion Reporter version 4.0) and independently using a pipeline constructed with Bowtie2 [24] and GATK [25]. The Bowtie2-GATK pipeline maps the raw reads of each library to hg19 using the default settings of Bowtie2. Variants were called on this set of reads using GATK's haplotypecaller. Alignments were visualized using the Integrative Genomics Viewer [26].

### Statistical analysis

Fisher's exact test for r×c tables was used to test for significance of associations between the parameters. All *p* reported are one-sided probabilities. All analysis was done using Stata version 11 (StataCorp LP).

## Results

### Immunohistochemistry and CISH

A total of 112 cases were analyzed by a TMA platform. In addition, 6 cases were analyzed by full section because these were more recent cases that had not been incorporated into the TMA.

Following ALK immunohistochemical staining, due to missing cores or the absence of tumor in the cores, complete results for all three antibodies were available for 105 cases. Following *ALK* CISH, in addition to missing cores or the absence of tumor in the cores, 12 cases were technically unsuccessful (no signal detected), while 2 cases had marked crush artefact resulting in interpretation difficulties. Therefore, complete results for all three antibodies and *ALK* CISH were available for 91 cases.

The IHC results for the three different antibodies are displayed in Table 4. The D5F3 antibody detected the most, while the ALK1 antibody detected the least, number of positive cases (percentage of positive cases for D5F3, 5A4 and ALK1 antibodies are 13.3% (14/105), 0.02% (2/105) and 0.01% (1/105) respectively.

**Table 4:** Sample type and ALK immunohistochemical expression (N = 105).

	ALK1 clone			5A4 clone				D5F3 clone			
**IHC Score**	0	1+	3+	0	1+	2+	3+	0	1+	2+	3+
**Pre-treatment primary tumors**	28	0	0	20	5	3	0	11	8	6	3
**Post-treatment primary tumors**	49	0	1	41	7	1	1	17	19	9	5
**Relapsed/metastatic tumors**	23	1	0	15	5	3	1	4	10	4	6
**Treatment unknown**	3	0	0	1	2	0	0	1	0	2	0
***p*** ** (Fisher**'**s exact test)**	0.519			0.165				0.322			

**Figure 1:**
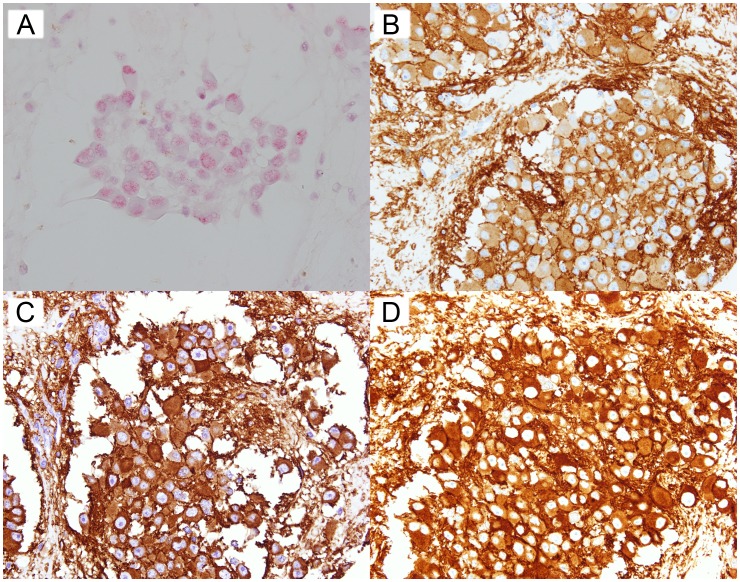
ALK CISH and IHC of an *ALK* amplified case. (A) CISH (B) ALK1 IHC (C) 5A4 IHC (D) D5F3 IHC.

**Figure 2:**
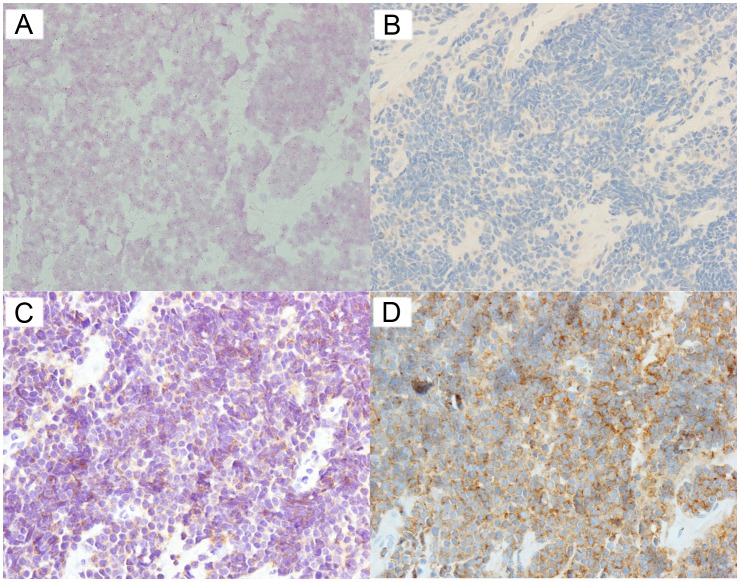
ALK CISH and IHC of an *ALK* non-amplified case. (A) CISH (B) ALK1 IHC (C) 5A4 IHC (D) D5F3 IHC.

We detected one *ALK* amplified (positive) case, with all the tumor cells in the TMA core displaying more than 6 *ALK* copy numbers/nucleus. Of note, this was the only case in our cohort displaying 3+ ALK1 IHC expression (Figure 1). The remaining cases were *ALK* non-amplified (negative); there were no cases with equivocal *ALK* amplification status. Figure 2 illustrates the ALK IHC profiles of an *ALK* non-amplified case.

Tables 5, 6 and 7 show the correlation between *ALK* CISH and ALK IHC for the three antibodies. There were statistically significant positive correlations between ALK1 IHC expression and *ALK* amplification status; and 5A4 IHC expression and *ALK* amplification status. Of note, the majority of ALK D5F3 IHC-positive neuroblastomas did not possess *ALK* genomic amplification.

**Table 5:** Correlation between ALK1 IHC and *ALK* CISH (N = 91).

	*ALK* amplified	*ALK* non-amplified
**ALK1 IHC 3+**	1	0
**ALK1 IHC 0–2+**	0	90

*p* (Fisher's exact test)  = 0.011.

**Table 6:** Correlation between 5A4 IHC and *ALK* CISH (N = 91).

	*ALK* amplified	*ALK* non-amplified
**5A4 IHC 3+**	1	1
**5A4 IHC 0–2+**	0	89

*p* (Fisher's exact test)  = 0.022.

**Table 7:** Correlation between D5F3 IHC and *ALK* CISH (N = 91).

	*ALK* amplified	*ALK* non-amplified
**D5F3 IHC 3+**	1	12
**D5F3 IHC 0–2+**	0	78

*p* (Fisher's exact test)  = 0.143.

### Sanger and IT-PGM sequencing

53 patient samples were subjected to Sanger sequencing of ALK exons 23 and 25 for the detection of p.F1174 and p.R1275 mutations. We identified 3 cases with the p.F1174L mutation, and 2 cases with the p.R1275Q mutation (Table 8). The correlation between the mutation status and D5F3 IHC expression is shown in Table 9.

**Table 8:** *ALK* mutations detected by both Sanger sequencing and IT-PGM.

Case Number	Coding DNA	Amino acid	Depth of Coverage (x)	Variant allele frequency (%)
11011	c.3522C>G	p.F1174L	1992	50.45
145808	c.3522C>A	p.F1174L	782	15.09
70309	c.3824G>A	p.R1275Q	897	20.07
180713	c.3824G>A	p.R1275Q	1536	41.99
184213	c.3522C>A	p.F1174L	1996	56.21

**Table 9:** Correlation between ALK D5F3 IHC score and mutation status.

	p.F1174L	p.R1275Q	p.F1174 and p.R1275 wild-type
**D5F3 IHC 3+**	1	2	5
**D5F3 IHC 2+**	2	0	11
**D5F3 IHC 1+**	0	0	21
**D5F3 IHC 0**	0	0	12

*p* (Fisher's exact test)  = 0.004.

**Figure 3:**
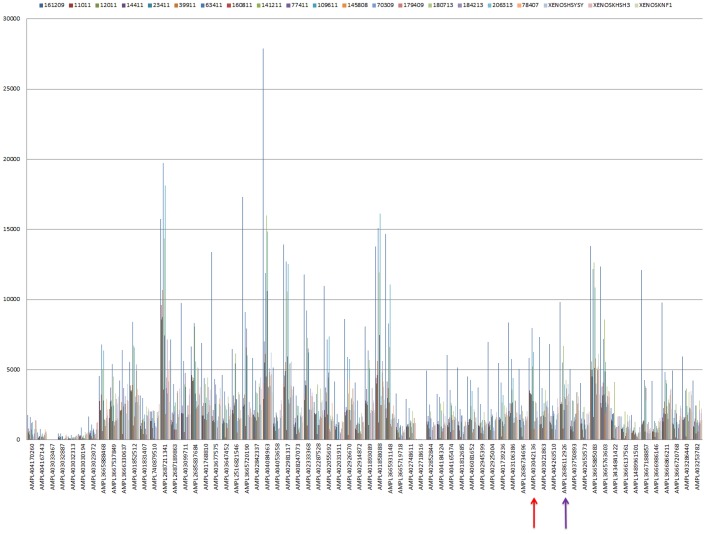
Depth of coverage of amplicons from 5′ to 3′. The p.F1174 and p.R1275 hotspots are covered by the amplicons marked with a red and purple arrow respectively.

18 patient samples and 3 cell line-derived tumor xenografts were analyzed by IT-PGM. 19 of 21 samples showed adequate uniformity of coverage, with less than 2% of bases with zero coverage. The other 2 cases (145808 and 180713) had around 30% of bases with zero coverage. The depth of coverage across the *ALK* coding region for all 21 samples is illustrated in Figure 3.

Of particular interest to us, the depth of coverage for the amplicons encompassing the p.F1174 and p.R1275 hotspots ranged from 741 to 7968× (AMPL403042136), and 590 to 5059× (AMPL403750893) respectively.

**Figure 4:**
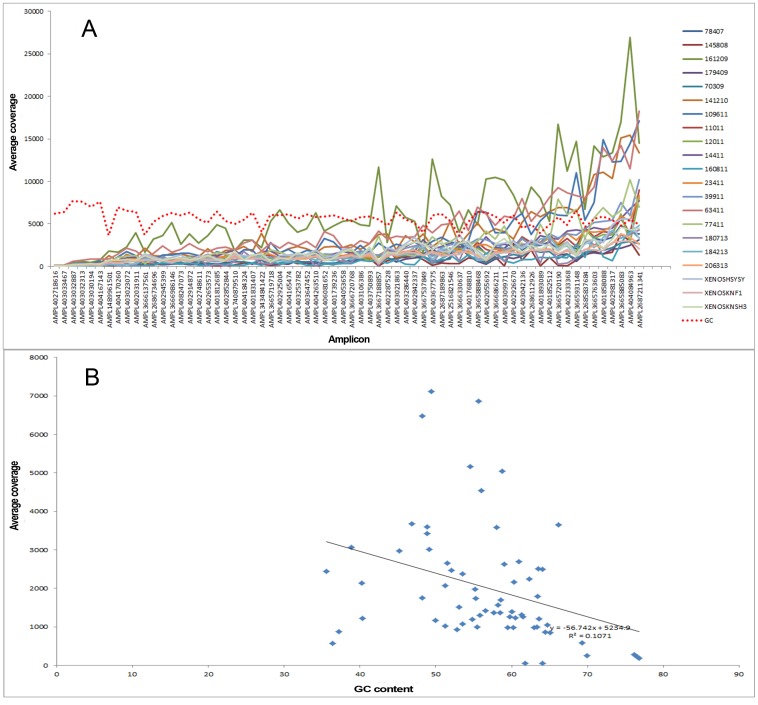
Depth of coverage and GC content. (A) The red dashed line indicates the GC content. The X-axis shows the amplicons ordered with increasing depth of coverage from left to right. (B) There is a negative correlation between depth of coverage and GC content; this is not statistically significant (R^2^ = 0.1).

We were interested to study the correlation between depth of coverage and GC content, as undercoverage of GC-rich regions during the PCR amplification step of the library construction is a reported phenomenon resulting in coverage bias [27], [28]. We found a negative correlation between the depth of coverage and GC content, although this was not statistically significant (R^2^ = 0.1) (Figure 4).

**Figure 5:**
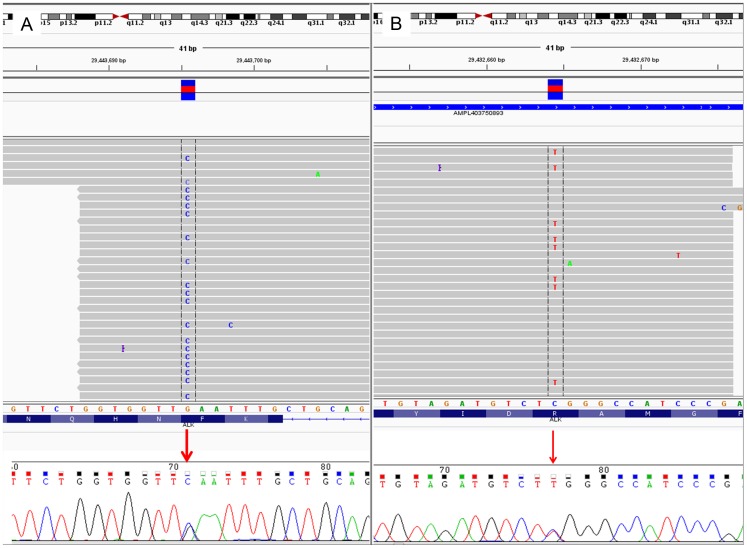
IGV (IT-PGM) and Sanger sequences of *ALK* hotspot mutations. (A) c.3522C>G (p.F1174L) mutation. (B) c.3824G>A (p.R1275Q) mutation.

There was a perfect correlation between the results obtained by IT-PGM and Sanger sequencing for all 18 patient samples (3 cases with p.F1174L; 2 cases with p.R1275Q; 13 wild-type) and 3 cell line-derived tumor xenografts (2 with p.F1174L; 1 wild-type). A representative case is illustrated in Figure 5. Of note, our mutational analyses for the xenografts corroborate with that reported in the literature: SKNF1 – ALK wild-type (WT); SH-SY5Y and SK-N-SH – p.F1174L) [14], [29].

Correlation between ALK D5F3 IHC and hotspot mutational analysis results revealed that the majority of ALK D5F3 IHC-positive neuroblastomas did not possess the hotspot mutations.

### Correlation of ALK IHC, copy number and mutation status with other clinicopathological parameters

We also correlated ALK IHC, copy number and mutation status with histology and *MYCN* copy number status (Tables S1, S2, S3, S4, S5, S6, S7, S8, S9 and S10 in File S1). We observed statistically significant positive correlations between ALK D5F3 IHC and *MYCN* copy number status; and *ALK* mutation status and *MYCN* copy number status (Tables S3 and S5 in File S1). No other statistically significant associations were observed.

## Discussion

The evaluation of various diagnostic platforms for ascertainment of ALK status is an important topic in lung carcinoma [30], [31], and this subject will become increasingly prominent in neuroblastoma diagnostics. Our paper is an early study on this emerging theme.

In the lung cancer literature, much attention has been devoted to the comparison of different ALK antibodies for immunohistochemistry [30], [32]. The earliest antibody developed was the ALK1 clone [33], which has primarily been used diagnostically in the detection of *ALK*-rearranged anaplastic large-cell lymphomas. In 2010, a comparison study by Mino-Kenudson et al. [34] reported that the D5F3 clone appeared to be more sensitive than ALK1 in detecting ALK-rearranged lung adenocarcinomas. At present, the exact mechanism for this increased sensitivity is not known. Based on recent publications, D5F3 appears to be a fairly popular choice for ascertainment of ALK protein expression status in lung carcinomas [35]–[37].

Unlike in lung carcinomas where the sensitivity and specificity of ALK IHC can be compared against *ALK* rearrangement status (as evaluated by FISH or CISH), such a ‘gold standard’ reference does not exist in neuroblastomas, since the mechanism for ALK protein expression does not appear to involve a genetic event in the majority of cases (as noted previously, the prevalence of strong ALK protein expression is generally 50% or higher, whereas the prevalence of *ALK* genomic amplification and/or mutations is around 10%). We were thus not able to establish sensitivity and specificity of the various antibodies.

However, we observed a statistically significant positive correlation between ALK D5F3 IHC and *MYCN* copy number status, which is consistent with the literature reporting a positive correlation between ALK gene/protein expression and *MYCN* amplification [5], [38]. Since *MYCN* amplification is a well known adverse prognostic marker in neuroblastomas [39], this provides further support that the ALK D5F3 antibody is likely the most suitable among the three for ascertaining ALK protein expression status as a prognostic biomarker. It will be necessary to evaluate this antibody in prospective clinical trials to robustly determine its clinical utility as a prognostic and predictive therapeutic biomarker.

We have also shown that a single color *ALK* CISH assay enables detection of *ALK* genomic amplification in neuroblastomas. The advantages of employing CISH instead of FISH are well known, and include convenience resulting from use of a brightfield instead of a fluorescence microscope; a greater ability to correlate gene copy numbers with morphological features; and the dispensation of a requirement for micrograph storage, necessary in the case of FISH because of the photolability of the probes [40].

At present, compared to ALK immunohistochemical expression, *ALK* copy number status has not been as widely studied as a biomarker in neuroblastomas, probably because of the rarity of *ALK* genomic amplification (prevalence 5% or less) [5], [7], [8]. However, it is worth noting that *ALK* genomic amplification correlates with a poor outcome [5], [7], [8], and that an *in vitro* drug sensitivity study revealed that an *ALK* amplified cell line (NB1) displayed enhanced sensitivity to growth inhibition by crizotinib [14]. Our study shows that single color CISH can be employed in future clinical trials to ascertain the *ALK* copy number profile and its utility as a prognostic and predictive biomarker.

We were able to successfully utilize a NGS platform for the detection of the *ALK* hotspot mutations (p.F1174L and p.R1275Q), which are present in up to 10% of neuroblastomas [7], [10]–[12]. Although NGS might not be the most cost-effective method for identification of only two mutations in an uncommon tumor, it becomes a more viable alternative to conventional Sanger sequencing if the region of interest covers most, or all, of the entire *ALK* gene. Rare *ALK* mutations, with at present uncertain clinical significance, are known to occur outside of regions covered by amplicons for exons 23 and 25, where the p.F1174 and p.R1275 hotspots are located. These include truncations involving exons 2–3 [29] or exons 4–11 [41], and mutations in exon 20 (e.g. p.D1091N) and exon 22 (e.g. p.T1151M) [42]. We envisage that future diagnostic panels encompassing multiple genes will be the most cost-effective way forward for exploiting an NGS platform for neuroblastoma diagnostics.

With regards to the IT-PGM sequencing, we observed several amplicons that showed low read depth (Figure 3). Amplicon read depth is an important, but to date not well-studied phenomenon, in NGS diagnostics. One of the well-known reasons that might account for low amplicon read depth, as mentioned above, is the presence of GC-extreme regions [27], [28]. However, this was not found to be the case in our study, as seen by the lack of significant statistical association between GC content and amplicon read depth. Based on our findings, optimization of the protocol, e.g. re-designing primers, will be necessary to achieve adequate amplicon read depth to the standard required for NGS diagnostics.

In conclusion, we have evaluated several platforms for the ascertainment of ALK status in neuroblastomas. Our study reveals the different staining properties of the commonly used ALK antibodies employed in routine diagnostics, in particular the increased sensitivity of the D5F3 antibody. It will be necessary for future clinical trials evaluating ALK as a prognostic and therapeutic predictive biomarker to determine the optimal antibody for routine diagnostics. This is particularly important for ALK IHC-positive but *ALK* copy number/mutation-negative neuroblastomas, in which the utility of ALK targeted therapeutics is at present unascertained. In addition, we find that single-color CISH and IT-PGM sequencing are suitable assays to ascertain *ALK* genomic copy and mutational status respectively, and these should also be employed and evaluated in future clinical trials.

## Supporting Information

File S1 **Tables S1–S10.** Table S1 in File S1. Correlation between ALK1 IHC and *MYCN* status (N = 96). Table S2 in File S1. Correlation between 5A4 IHC and *MYCN* status (N = 98). Table S3 in File S1. Correlation between D5F3 IHC and *MYCN* status (N = 97). Table S4 in File S1. Correlation between *ALK* CISH and *MYCN* status (N = 83). Table S5 in File S1. Correlation between *MYCN* status and *ALK* mutation status (N = 48). Table S6 in File S1. Correlation between ALK1 IHC and histology (N = 104). Table S7 in File S1. Correlation between 5A4 IHC and histology (N = 106). Table S8 in File S1. Correlation between D5F3 IHC and histology (N = 105). Table S9 in File S1. Correlation between *ALK* CISH and histology (N = 91). Table S10 in File S1. Correlation between *ALK* mutational status and histology (N = 54).
(DOCX)
