# Supplementary material for: Platform Comparison for Evaluation of ALK Protein Immunohistochemical Expression, Genomic Copy Number and Hotspot Mutation Status in Neuroblastomas
Source: PLoS One. 2014 Sep 4;9(9):e106575. doi: 10.1371/journal.pone.0106575 (PMC4154751; doi:10.1371/journal.pone.0106575)
Supplement: File S1 — Tables S1–S10. Table S1 in File S1. Correlation between ALK1 IHC and MYCN status (N = 96). Table S2 in File S1. Correlation between 5A4 IHC and MYCN status (N = 98). Table S3 in File S1. Correlation between D5F3 IHC and MYCN status (N = 97). Table S4 in File S1. Correlation between ALK CISH and MYCN status (N = 83). Table S5 in File S1. Correlation between MYCN status and ALK mutation status (N = 48). Table S6 in File S1. Correlation between ALK1 IHC and histology (N = 104). Table S7 in File S1. Correlation between 5A4 IHC and histology (N = 106). Table S8 in File S1. Correlation between D5F3 IHC and histology (N = 105). Table S9 in File S1. Correlation between ALK CISH and histology (N = 91). Table S10 in File S1. Correlation between ALK mutational status and histology (N = 54). (DOCX) [file pone.0106575.s001.docx]

**Supporting Information**

**Table S1 Correlation between ALK1 IHC and *MYCN* status (N = 96)**

|  | ***MYCN* non-amplified** | ***MYCN* equivocal** | ***MYCN* amplified** |
| --- | --- | --- | --- |
| **ALK1 IHC 0-2+** | 75 | 1 | 19 |
| **ALK1 IHC 3+** | 0 | 0 | 1 |

*p* (Fisher’s exact test) = 0.219

**Table S2 Correlation between 5A4 IHC and *MYCN* status (N = 98)**

|  | ***MYCN* non-amplified** | ***MYCN* equivocal** | ***MYCN* amplified** |
| --- | --- | --- | --- |
| **5A4 IHC 0-2+** | 77 | 1 | 19 |
| **5A4 IHC 3+** | 0 | 0 | 1 |

*p* (Fisher’s exact test) = 0.214

**Table S3 Correlation between D5F3 IHC and *MYCN* status (N = 97)**

|  | ***MYCN* non-amplified** | ***MYCN* equivocal** | ***MYCN* amplified** |
| --- | --- | --- | --- |
| **D5F3 IHC 0-2+** | 74 | 0 | 10 |
| **D5F3 IHC 3+** | 3 | 1 | 9 |

*p* (Fisher’s exact test) <0.001

**Table S4 Correlation between *ALK* CISH and *MYCN* status (N = 83)**

|  | ***MYCN* non-amplified** | ***MYCN* equivocal** | ***MYCN* amplified** |
| --- | --- | --- | --- |
| ***ALK* non-amplified** | 66 | 1 | 15 |
| ***ALK* amplified** | 0 | 0 | 1 |

*p* (Fisher’s exact test) = 0.205

**Table S5 Correlation between *MYCN* status and *ALK* mutation status (N = 48)**

|  | ***MYCN* non-amplified** | ***MYCN* equivocal** | ***MYCN* amplified** |
| --- | --- | --- | --- |
| **p.F1174 and p.R1275 wild-type** | 39 | 1 | 4 |
| **p.F1174L** | 0 | 0 | 2 |
| **p.R1275Q** | 0 | 0 | 2 |

*p* (Fisher’s exact test) = 0.001

**Table S6 Correlation between ALK1 IHC and histology (N = 104)**

|  | **Ganglioneuroblastoma** | **Ganglioneuroma** | **Neuroblastic tumor, unspecified** | **Neuroblastoma** |
| --- | --- | --- | --- | --- |
| **ALK1 IHC 0** | 15 | 3 | 29 | 55 |
| **ALK1 IHC 1+** | 0 | 0 | 1 | 0 |
| **ALK1 IHC 3+** | 0 | 0 | 0 | 1 |

*p* (Fisher’s exact test) = 0.712

**Table S7 Correlation between 5A4 IHC and histology (N = 106)**

|  | **Ganglioneuroblastoma** | **Ganglioneuroma** | **Neuroblastic tumor, unspecified** | **Neuroblastoma** |
| --- | --- | --- | --- | --- |
| **5A4 IHC 0** | 13 | 3 | 21 | 43 |
| **5A4 IHC 1+** | 2 | 0 | 6 | 11 |
| **5A4 IHC 2+** | 0 | 0 | 3 | 2 |
| **5A4 IHC 3+** | 0 | 0 | 0 | 2 |

*p* (Fisher’s exact test) = 0.852

**Table S8 Correlation between D5F3 IHC and histology (N = 105)**

|  | **Ganglioneuroblastoma** | **Ganglioneuroma** | **Neuroblastic tumor, unspecified** | **Neuroblastoma** |
| --- | --- | --- | --- | --- |
| **D5F3 IHC 0** | 7 | 3 | 11 | 13 |
| **D5F3 IHC 1+** | 6 | 0 | 9 | 23 |
| **D5F3 IHC 2+** | 2 | 0 | 6 | 12 |
| **D5F3 IHC 3+** | 0 | 0 | 4 | 9 |

*p* (Fisher’s exact test) = 0.284

**Table S9 Correlation between *ALK* CISH and histology (N = 91)**

|  | **Ganglioneuroblastoma** | **Ganglioneuroma** | **Neuroblastic tumor, unspecified** | **Neuroblastoma** |
| --- | --- | --- | --- | --- |
| ***ALK* non-amplified** | 15 | 1 | 23 | 51 |
| ***ALK* amplified** | 0 | 0 | 0 | 1 |

*p* (Fisher’s exact test) = 1.00

**Table S10 Correlation between *ALK* mutational status and histology (N = 54)**

|  | **Ganglioneuroblastoma** | **Ganglioneuroma** | **Neuroblastic tumor, unspecified** | **Neuroblastoma** |
| --- | --- | --- | --- | --- |
| **p.F1174 and p.R1275 wild-type** | 9 | 0 | 12 | 28 |
| **p.F1174L** | 0 | 0 | 2 | 1 |
| **p.R1275Q** | 0 | 0 | 0 | 2 |

*p* (Fisher’s exact test) = 0.466
